# High sensitivity Troponin-I levels in asymptomatic hemodialysis patients

**DOI:** 10.1080/0886022X.2019.1603110

**Published:** 2019-05-28

**Authors:** Tanawat Tarapan, Khrongwong Musikatavorn, Piyarat Phairatwet, Kullaya Takkavatakarn, Paweena Susantitaphong, Somchai Eiam-Ong, Khajohn Tiranathanagul

**Affiliations:** a Emergency Medicine Unit, Outpatient Department, King Chulalongkorn Memorial Hospital, The Thai Red Cross Society, Bangkok, Thailand;; b Emergency Medicine Unit, Department of Medicine, Faculty of Medicine, Chulalongkorn University, Bangkok, Thailand;; c Bhumirajanakarindra Kidney Institute Hospital, Bangkok, Thailand;; d Division of Nephrology, Department of Medicine, King Chulalongkorn Memorial Hospital, Thai Red Cross Society and Faculty of Medicine, Chulalongkorn University, Bangkok, Thailand

**Keywords:** Hemodialysis, Troponin I, myocardial injury

## Abstract

Reduction in renal clearance and removal by hemodialysis adversely affect the level and utility of high-sensitivity troponin I (hsTnI) for diagnosis of acute myocardial infarction (AMI) in hemodialysis (HD) patients. Furthermore, HD process itself might cause undesirable myocardial injury and enhance post HD hsTnI levels. This comparative cross-sectional study was conducted to compare the hsTnI levels between 100 asymptomatic HD patients and their 107 matched non-chronic kidney disease (CKD) population. The hsTnI levels in HD group were higher than non-CKD group [median (IQR): 54.3 (20.6–152.7) vs. 18 (6.2–66.1) ng/L, *p* < .001)]. The hsTnI levels reduced after HD process from 54.3 (20.6–152.7) ng/L in pre-HD to 27.1 (12.3–91.4) ng/L in post-HD (*p* = .015). Of interest, 25% of HD patients had increment of hsTnI after HD and might represent HD-induced myocardial injury. The significant risk factors were high hemoglobin level and high blood flow rate. In conclusion, the baseline hsTnI levels in asymptomatic HD patients were higher than non-CKD population. The dynamic change of hsTnI over time would be essential for the diagnosis of AMI. Certain numbers of asymptomatic HD patients had HD-induced silent myocardial injury and should be aggressively investigated to prevent further cardiovascular mortality.

## Introduction

Early precise diagnosis of acute myocardial infarction (AMI) leads to appropriate treatment and better clinical outcome. Besides clinical symptoms and electrocardiogram, elevated cardiac biomarkers, especially human cardiac troponin I (TnI, 209 amino acids and molecular weight of 23,875 Dalton) [1,2] above the reference cutoff point derived from general population, are crucial parameters in diagnosis of AMI [3–5].

Growing number of hemodialysis (HD) patients has been observed around the world. HD patients have higher risks for developing AMI than general population [6]. It is still problematic in using TnI in diagnosis of AMI in HD patients because reduction in renal clearance and potential removal by modern HD techniques can adversely affect the level and utility of this marker in HD patients. There were uncertainties in previous data of plasma TnI levels measured by conventional assays in asymptomatic HD patients compared with the cutoff point derived from general population [7–11].

Indeed, conventional assays of TnI levels used in most previous studies were not sensitive enough to detect the accurate TnI levels. A modern TnI measurement, called ‘high-sensitivity TnI; hsTnI’, provided higher sensitivity in detecting very low levels of TnI [12]. The reference range of hsTnI was established from the study in general healthy population (18–64 year-old) without hypertension, diabetes, chronic kidney disease (CKD), and myocardial infarction [13]. The suggested cutoff points of hsTnI from the study were 36 ng/L in men (*n* = 272) and 15 ng/L in women (*n* = 252) [13].

There were few studies using hsTnI assay in HD patients. The percentage of asymptomatic HD patients who had pre-dialysis hsTnI levels higher than the reference cutoff point were 5–51% [14–19]. Studies regarding the effect of HD on TnI levels, measured by conventional assays, are contradictory [20,21]. The studies that used the newer hsTnI assay still provided limited data [14–19]. In one study, low-flux dialyzer was used [17]. This might not represent the majority of HD patients currently undergoing high-flux HD which includes either HD or online hemodiafiltration (HDF) using high-flux dialyzer. Three studies in high-flux HD patients included quite a small number of patients [14,18,19] while the other two studies did not focus on the subgroup of patients with heightened post-dialysis hsTnI from possible intradialytic myocardial hypoperfusion [15,16]. On the contrary, modern HD modalities might remove TnI molecule and reduce post-dialysis hsTnI levels. Of interest, all of the above studies focused on a single group of dialysis patients without matched non-CKD control. Therefore, the factors other than HD and CKD that might affect the hsTnI levels could not be totally excluded.

The present study was conducted to compare the hsTnI levels in asymptomatic high-flux HD patients with the non-CKD population as the primary outcome. The changes of hsTnI after dialysis were evaluated to determine intradialytic hsTnI changes and the subgroup of patients with increased post HD hsTnI levels. The factors that might be associated with the elevated post HD hsTnI levels were also assessed.

## Patients and methods

### Patients

This cross-sectional study was conducted in 100 asymptomatic chronic high-flux HD patients in two participated hospitals and 107 non-CKD population. The study was approved by the Institutional Review Boards of Faculty of Medicine, Chulalongkorn University and Bhumirajanagarindra Kidney Institute hospital, Bangkok, Thailand. All studied subjects provided written consent for collection of the blood samples and participation in the study. The asymptomatic ESRD patients receiving high-flux HD which included either HD or online HDF using high-flux dialyzer at King Chulalongkorn Memorial Hospital and Bhumirajanagarindra Kidney Institute hospital were enrolled in the study. The standard dialysis fluid and substitution fluid compositions prescription were sodium 138 mEq/L, calcium 2.5 mEq/L, potassium 2 mEq/L, and bicarbonate 32 mEq/L. The inclusion criteria for asymptomatic HD patients comprised age older than 18 years old, dialysis vintage more than 1 year, thrice-a-week schedule, and no active cardiovascular disease during the past 6 months. Demographic data, past medical history, and laboratory parameters were obtained from the patients and medical records.

The asymptomatic non-CKD group was enrolled from the volunteers with Egfr > 60 mL/min/1.73 m^2^ who came to outpatient department for scheduled follow up. The non-CKD subjects with any events of AMI, heart failure, or sepsis during three months prior to the study were excluded.

### Sampling collection and measurement

Blood samples before and after mid-week high-flux HD in the study group and a single blood sample in the non-CKD group were collected. The blood samples for hsTnI determination were stored at −20 °C and later analyzed by ARCHITECT STAT High Sensitive Troponin-I assay (Abbott Diagnostics), a chemiluminescence microparticle immunoassay (CMIS). The pre-dialysis blood sample was immediately analyzed for complete blood count (CBC) and other biochemical parameters whereas the post-dialysis blood sample was tested for CBC. The pre- and post-dialytic hematocrit (Hct) values were used for correcting post-dialytic hsTnI concentrations from ultrafiltration-induced hemoconcentration.

### Statistical analysis

All analyses were performed using SPSS 17.0 software system (SPSS, Chicago, IL, USA). The continuous data were reported as mean ± SD or median (25th to 75th percentiles, interquartile range, IQR). The nominal data were presented as percentages.

The comparisons between continuous variables were performed by Student’s t-test for normal distribution data and the Mann–Whitney *U* test or McNemar’s test for non-normal distribution data. The chi-square test was used to compare the proportion of variables between the study group and the non-CKD group as well as between sub-groups of intradialytic hsTnI reduction and hsTnI elevation in HD study groups for univariate analysis. The multivariable logistic regression analysis was performed by including significant factors in univariate analysis to identify the factors that are independently associated with the hsTnI elevation sub-group. The differences were considered significant at *p* < .05.

## Results

One hundred asymptomatic high-flux HD patients (HD group) and 107 asymptomatic non-CKD volunteers (control group) were enrolled in the present study. The demographic data including age, gender, and body weight were comparable (Table 1).

**Table 1.** Demographic and clinical characteristic data of non-CKD and HD groups.

	Non-CKD *n* = 107	HD *n* = 100	*p-value*
Age [mean, range (year)]	63.8 ± 9.97, 43–86	64.5 ± 13.8, 23–92	*NS*
Male gender [*n* (%)]	41 (38.3%)	44 (44%)	*NS*
Body weight (Kg)	62.4 ± 10.2	57.1 ± 12.8	*NS*
Co-morbid diseases [*n* (%)]			
Diabetes mellitus	51 (47.7%)	49 (49%)	*NS*
Hypertension	78 (72.9%)	82 (82%)	*NS*
Cerebrovascular disease	3 (2.8%)	7 (7%)	*NS*
Coronary artery disease	9 (8.4%)	14 (14%)	*NS*
with PCI	6 (5.6%)	8 (8%)	*NS*

PCI: percutaneous coronary intervention.

The HD group was on dialysis (dialysis vintage) for 4.5 (1–7) years. The current modalities were HD (*n* = 75, 75%) and post-dilution online HDF (*n* = 25, 25%). Types of vascular access included 80 (80%) for AV fistula, 16 (16%) for AV graft, and 4 (4%) for permanent cuffed catheter. The blood flow and dialysis fluid flow were 358.1 ± 47.5 and 718.2 ± 133.6 mL/min, respectively. The substitution volume was 23.1 ± 1.2 L in post-dilution online HDF (*n* = 25). Their Kt/V values were 2.09 ± 0.45 (2.07 ± 0.47 vs. 2.28 ± 0.37, *p* = .08 for HD and online HDF, respectively).

### Pre-dialysis hsTnI

**Figure 1.**
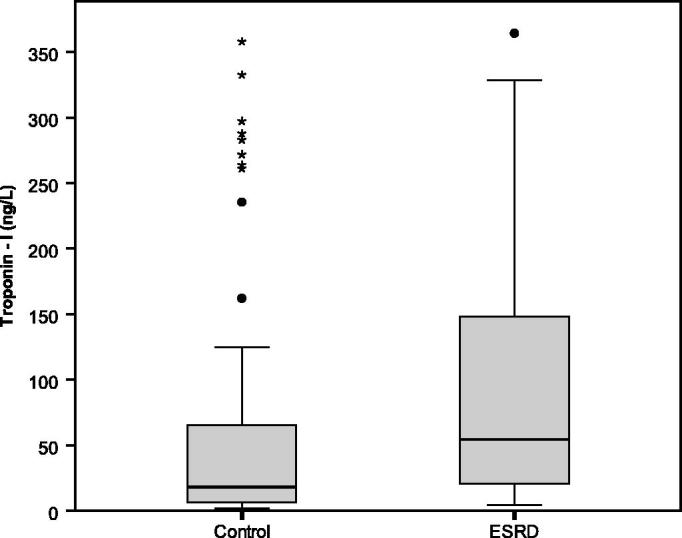
The baseline of hsTnI in asymptomatic non-CKD group and pre-dialysis HD group as well as post-dialysis in HD group.

The HD group had significantly higher hsTnI levels than the non-CKD group (Figure 1) with median values of 54.3 (20.6–152.7) vs 18 (6.2–66.1) ng/L (*p* < .05), respectively. In HD group, there was no significant difference between subgroups of HD and HDF.

The reference cutoff points of 36 ng/L in male and 15 ng/L in female were proposed from the previous study as a normal range in general population [13]. Pre-dialysis hsTnI levels above the cutoff point were present in 73 patients (73%, 26/44 in male and 47/56 in female) whereas 49 of the non-CKD control group (45%, 13/41 in male and 36/66 in female) had high hsTnI (*p* < .01).

### Intra-dialytic changes in hsTnI

The pre-dialysis hsTnI levels were significantly higher than post-dialysis values [54.3 (20.6–152.7) vs. 27.1 (12.3–91.4) ng/L, *p* = .015] (Figure 1). The median percentage of reduction were 36 (8.3–75)%.

Of one hundred HD patients, there were 25 patients (25%) with the post-dialytic hsTnI levels higher than pre-dialysis values. Their post-dialytic hsTnI changes were +589.7 ± 823.9 ng/L compared with −95.6 ± 142.3 ng/L for the remaining (*p* < .001). There was no clinical chest pain documented in this group of patients. This asymptomatic raising of hsTnI level instead of reduction by dialytic clearance might represent the silent myocardial injury during hemodialysis. The potential factors that might affect this hsTnI elevation group were analyzed and compared with the hsTnI reduction group. Five demographic and clinical factors were significantly identified as the risk factors after univariate analysis including age, body weight, hemoglobin, blood flow, and dialysate flow. The demographic and laboratory parameters between these two groups were demonstrated in Table 2. The multivariate linear regression analysis was performed using these factors. Only Hb higher than 11 g/dL (*p* = .013) and dialysate flow more than 500 mL/min (*p* = .011) were identified as the independent factors associated with hsTnI elevation sub-group.

**Table 2.** Comparison between HD patients with post-dialysis hsTnI reduction and elevation subgroup.

	hsTnI reduction (*N* = 75)	hsTnI elevation (*N* = 25)	*p* value
Gender (Male)	30 (41%)	14 (56%)	NS
Age (year)	66.15 ± 12.9	59.80 ± 15.4	<.05
Body weight (kg)	55.76 ± 12.5	61.84 ± 12.9	<.05
Diabetes mellitus	36 (49%)	13 (52%)	NS
Hypertension	61 (82%)	21 (84%)	NS
History of stroke	5 (7%)	2 (8%)	NS
Ischemic heart disease	11 (15%)	3 (12%)	NS
History of percutaneous coronary intervention	6 (8%)	1 (4%)	NS
Clinical parameters			
Systolic BP (mmHg)	158 ± 27	158 ± 17	NS
Diastolic BP (mmHg)	70 ± 15	71 ± 13	NS
Pulse (beats/min)	72 ± 13	70 ± 11	NS
Hemodialysis parameters			
Dialysis vintage (years)	4.5 ± 5	4.8 ± 4.8	NS
Blood flow (mL/min)	352 ± 49	376 ± 38.5	<.05
Dialysate flow (mL/min)	701 ± 141	776 ± 83	<.05
Weight gain (%)	4.6 ± 2.2	4.7 ± 1.9	NS
Weight loss (%)	3.9 ± 1.5	4.1 ± 1.4	NS
Biochemical parameters			
Pre-dialysis hsTnI (ng/L)	57.5 (26.2–157.3)	24 (10.2–141.4)	NS
Hematocrit (%)	33 ± 4.4	34 ± 3.4	NS
Hemoglobin (g/dL)	10.5 ± 1.2	11.1 ± 1.1	<.05
HbA1C (%)	5.7 ± 1.3	6.6 ± 2	NS
Cholesterol (mg/dL)	155 ± 34	159 ± 36	NS
Triglyceride (mg/dL)	107 ± 61	124 ± 65	NS
Albumin (g/dL)	3.8 ± 0.3	3.9 ± 0.4	NS

NS: not significant.

## Discussion

Elevated hsTnI plays a crucial role in diagnosis of AMI as defined by the major clinical guidelines and recommendations [3–5]. However, the levels might be affected by several factors especially the body clearance by the kidney and dialysis. The hsTnI usage in patients with certain special groups, particularly ESRD patients treated with long-term HD also provides limited information. Although hsTnI assay was utilized to provide more accurate result than the conventional assay, the suggested cutoff point for diagnosis of AMI was derived from normal population study [13]. Despite some recent studies had measured hsTnI levels in HD patients, there was no matched non-CKD group (Table 3)[14–19].

**Table 3.** Summary of studies of high sensitivity troponin-I level in asymptomatic chronic hemodialysis patients.

Authors (year, country)	Study design			HD modality in study group	Dialysis vintage (year)	Pre-dialysis hsTnI		Post-dialysis hsTnI	
	Design	Study group *(n*)	Control group (*n*)			Level (ng/L)	Patients with elevated hsTnI (%)	Intra-dialytic changes	Subgroup of heighted hsTnI
Assa et al. [17] (2013, Netherlands)	prospective	90	no	Low-flux HD, BFR 250–300 mL/min	3.5 (IQR 1.5–5.5)	20 (IQR 11–38)	34%	Increase	66% of patients
Kumar et al. [14] (2011, USA)	prospective	51	no	High-flux HD, BFR: unavailable	unavailable	25 (range 0–461)	37%	Not significant (n = 49)	Not performed
Gaiki et al. [15] (2012, USA)	prospective	51	no	High-flux HD, BFR: unavailable	–	No data	51%	–	–
Artunc et al. [16] (2012, German)	cross-sectional	239	no	High-flux HD (92%), BFR 300 mL/min	3.8 (IQR 1.6–7.1)	14 (IQR 7–29)	14%	–	–
Cardinaels et al. [18] (2015, Netherlands)	cross-over study	13	no	High-flux HD (at baseline)	4.1 ± 2.4	No data	23%	Not significant	Not performed
Skadberg et al. [19] (2016, Norway)	prospective	20	no	High-flux HD, BFR: unavailable	1.2 (range 0.2–9.6)	13.3 (range 3.9–115.9)	40%	Decrease (−7.6%)	5% (1/20) of patients
The present study (Tarapan et al.)	cross-sectional	100	107 non-CKD	High-flux HD, BFR 358 mL/min	4.5 (1–7)	54.3 (IQR 20.6–152.7)	73%	Decrease (−36%)	25% (25/100) of patients

As seen in Table 1, non-CKD status is the only different variable between the non-CKD and HD groups. The present study clearly demonstrated that hsTnI levels in HD patients were approximately three times significantly higher than the matched non-CKD population (Figure 1). As such, interpretation of hsTnI in HD patients should be performed cautiously since a single elevation of hsTnI may not indicate AMI [20]. Therefore, the elevation of hsTnI levels from the serial measurement should be used for the final diagnosis of AMI in HD patients.

The median pre-dialysis hsTnI levels in asymptomatic HD patients reported in previous studies were 13–25 ng/L (Table 3)[14,16,17,19]. The present study had the median pre-dialysis hsTnI levels of 54.3 ng/L (20.6–152.7). The percentage of asymptomatic HD patients in the present study who had higher pre-dialysis hsTnI levels than the reference cutoff point were 73% (26/44 in male and 47/56 in female), which were in the same trend with recent studies using hsTnI assay (Table 3)[14–19] and were higher than the reports that used conventional TnI assays. Therefore, the uncertainty in the issue of TnI elevation in asymptomatic HD patients could be answered by this modern high sensitive method. The heightened hsTnI levels might possibly result from the impaired residual renal clearance of hsTnI by the kidney and left ventricular hypertrophy (LVH) or dysfunction in HD patients. These factors were reported to enhance hsTnI levels and the effect might be more obvious along dialysis vintage [14,16]. As seen in Table 3, patients in the present study had longest dialysis vintage which might possibly cause higher pre-dialysis hsTnI levels than other previous studies. Another postulated cause of heightened hsTnI was related to bacteremia especially in patients with tunneled HD catheters [22]. However, there was only four percent of patients using catheters in the present study that could not affect the overall outcome. Indeed, earlier studies demonstrated that the high pre-dialysis hsTnI levels in asymptomatic HD patients could be a predictor of long-term mortality [15,17,23].

In the present study, there was quite a high number of population (45%) in the non-CKD group who had higher hsTnI levels than the reference cutoff point derived from general healthy population. The underlying cardiovascular-related conditions, such as hypertension, LVH, and peripheral arterial disease, might induce hsTnI elevation in the non-CKD group.

The effect of HD on hsTnI levels are unestablished, reporting either increasing [17], unchanged [14,18] or decreasing of post-dialysis hsTnI levels (Table 3) [19,24]. The present study demonstrated that dialysis process affected the levels of hsTnI. Post-dialysis levels were 36 (8.3 – 75)% lower than pre-dialysis values (Figure 1). These overall reductions of hsTnI after hemodialysis were greater than some previous studies [14,17–19]. One of the previous studies used low-flux HD which could not effectively remove large molecule like TnI[17]. The remaining studies that used high-flux HD reported median percentage reduction only up to 7.6%. This might be caused by the prescriptions of blood flow and dialysate flow rates in previous studies was lower than the present study [18,19]. The clearance by dialysis would be the explanation of this reduction. The molecular weight of TnI was 23,875 Dalton which could also be partially removed via convection by high-flux HD modality used in the present study. Besides this clearance mechanism, the adsorptive ability of the dialyzer membrane might also play a significant contribution. A previous *in-vitro* study found that the adherence of TnI to dialyzer membrane was responsible for the decrease in hsTnI after HD [24].

In previous studies, the incidence of elevated post-dialysis TnI was 66% in the patients with low-flux HD [17] and 5% with high-flux HD (Table 3) [19]. The majority of the patients (75%) in the present study demonstrated the reduction of hsTnI after dialysis (Table 2). The incidence of patients who had elevated post-dialysis TnI in the present study was 25% which was lower than a previous low-flux study [17] but was higher than an earlier high-flux study [19]. The elevated post-dialysis hsTnI group (25%) in the present study without clinical hemodynamic instability might indicate the real incidence of silent myocardial injury during high-flux HD. The levels of pre-dialysis hsTnI in the next session returned to comparable levels with the pre-dialysis of the study session (data not shown). These would confirm the impact of dialysis. Previous studies could also demonstrate this event by using various indicators including cTn, echocardiography, and cardiac MRI [25,26]. The coronary hemodynamic compromise during HD with ultrafiltration, even without obvious systemic hypotension, was postulated to be the explanation [20,21]. However, the present study did not find the difference in weight reduction volume parameters (Table 2). This present study prescribed DW by clinical assessment. As such, the issue of under DW could not be identified. It would be interesting to utilize other DW assessment methods especially bioimpedance method in this setting [27,28]. Unfortunately, the interesting long-term cardiac outcomes of this subgroup of patients could not be demonstrated in the present study because of the cross-sectional design and need further prospective study.

In the present study, the independent risk factors associated with post-dialysis hsTnI elevation included high hemoglobin level and high dialysate flow rate in multivariate analysis whereas the high blood flow rate was significant only in univariate analysis. Actually, the dialysate flow rate is usually prescribed following the blood flow rate and could be grouped together. Of interest, the elevated hsTnI group had higher hemoglobin level than the reduced hsTnI group (Table 2). This finding might relate to the issue of hemoglobin target in ESRD patients. Recently, several RCT studies on Hb target in pre-dialysis CKD and HD patients also demonstrated this observation [29–31]. The higher Hb target might enhance mortality rate or non-fatal MI in HD patients [29]. This adverse outcome of high Hb target might be more obvious in elderly HD patients [32]. As such, silent myocardial ischemia during dialysis might be one of the explanations. The current recommendation of lower Hb target than the level in the past would be supported by the finding in the present study.

By theory, the blood and dialysate flow rates should not affect the hemodynamics during hemodialysis. However, the high dialysate and/or substitution flow rate in online HDF and high-flux HD would relate to the sudden high bicarbonate influx and might partly contribute to this adverse event [33]. Therefore, the slow blood and dialysate flow rates were still one of the interventions utilized in case of intradialytic hypotension. The finding in the present study which is in agreement with some previous studies raises a consideration that the high blood flow rate might relate to the silent myocardial ischemia [16,34]. Nevertheless, some studies could not demonstrate this association [21,35].

Interestingly, the low Hb target and low blood, as well as dialysate flow strategies, are generally applied in Japanese dialysis practice which provides the highest survival rate when compared with other regions [36]. The major limitation of the present study was the small number of study population to confirm this sub-group analysis. Whether both strategies can actually lower the silent myocardial ischemia needs further larger study.

In conclusion, hsTnI levels in patients with ESRD receiving high-flux HD was significantly higher than non-CKD population with similar age, sex, and medical comorbidities. Therefore, CKD is one of the most important factors causing higher levels of hsTnI. High-flux HD could reduce the hsTnI levels by diffusive, convective, and adsorptive clearances. A certain number of patients might have silent myocardial ischemia which is possibly related to the high Hb level and high blood and dialysate flow rate, both of which should be concerned and further investigated.

## Disclosure statement

No potential conflict of interest was reported by the authors.
